# Editorial: Innovative organoid co-culture systems for enhanced precision medicine in cancer and beyond

**DOI:** 10.3389/fmed.2026.1871721

**Published:** 2026-05-27

**Authors:** Talita Glaser, Rupert Ecker, Uwe Ritter

**Affiliations:** 1 Department of Microbiology, Institute of Biomedical Sciences, University of São Paulo, São Paulo, Brazil; 2 Faculty of Health, School of Biomedical Sciences, Queensland University of Technology, Brisbane, QLD, Australia; 3 TissueGnostics GmbH, Vienna, Austria; 4 Chair for Immunology, University of Regensburg, Regensburg, Germany; 5 Leibniz Institute for Immunotherapy (LIT), Regensburg, Germany

**Keywords:** co-culture, *ex vivo* therapeutic response, organoid, personalized medicine, tumor microenvironment

## Introduction

Complex organoid co-culture systems that resemble tissue microenvironments are emerging as a powerful model for precision-medicine. Despite intrinsic *in vitro* limitations, patient-derived three-dimensional models closely recapitulate tissue architecture, cellular heterogeneity, and microenvironmental interactions. Organoid platforms integrate stromal, vascular, microbial, and immune elements, therefore, supporting mechanistic studies of disease, preclinical assessment of therapeutics, and individualized *ex-vivo* prediction of treatment response. However, clinical translation remains challenging based on methodological obstacles, particularly those related to components of vascular and immune integration. Computer-assisted analysis of organoid systems—be such analysis deterministic (i.e., classical image analysis) or AI-based—remains a critical bottleneck in the road map to full exploitation of what organoid co-culture systems might be able to deliver. The contributions in this Research Topic delineate a translational trajectory from mechanistic studies of barrier biology to engineered *ex vivo* immunotherapy assays and to organoid development for non-oncologic tissue repair. Collectively, they demonstrate the field's potential to advance precision medicine while exposing enduring methodological, bioengineering, and validation challenges that must be addressed for clinical translation.

## From barrier integrity to therapeutic resistance: organoid approaches to IFN-I (edited by Talita Glaser)

Type I interferons can help or harm the gut associated functionality. Therefore, detailed studies are needed to separate protective effects from damaging ones in inflammatory bowel disease (IBD). King et al. show that patient-derived intestinal organoids can offer a physiologically relevant platform to interrogate epithelial-specific IFN-I signaling. With addition of immune and microbial components, these models might enable systematic comparison of transient vs. sustained IFN-I exposure, allowing the assessment of effects on tight-junction integrity, epithelial proliferation, apoptosis, cellular turnover, and reparative programs. The authors prioritize epithelial barrier assays with immune and microbial partners to model signaling driving treatment failure. King et al. note that that interspecies differences between murine and human organoids can alter interferon-stimulated gene expression and cytokine sensitivity, a consideration that should inform experimental design and translational interpretation.

## Predicting anti-PD-1 response in colorectal cancer using patient-derived microtumors (edited by Talita Glaser)

Nguyen et al. describe a media-perfused 3D *ex vivo* platform that co-cultures patient-derived colorectal microtumors with autologous peripheral blood mononuclear cells (PBMCs) to functionally screen PD-1 blockade. The system preserves tissue architecture, endogenous tumor-infiltrating lymphocytes (TILs) and viability, and allows interstitial immune migration within bioconjugated liquid-like solid microgels. Using interferon-gamma (IFN-γ) measurements, confocal/TUNEL imaging, multiplexed ion beam imaging (MIBI), and whole-exome sequencing (WES), they report an MSI (microsatellite-instable) case with robust IFN-γ induction, immune infiltration and tumor apoptosis after pembrolizumab; an MSS (microsatellite-stable) case harboring a DNA polymerase epsilon (POLE) A456P exonuclease mutation with ultrahigh tumor mutational burden (TMB; ≈ 236 muts/Mb) showing *ex vivo* PD-1 sensitivity, particularly in microtumor-only cultures; and heterogeneous outcomes among other POLE-mutated tumors, underscoring variant pathogenicity and the importance of spatial immune context. The observed attenuation of response upon adding autologous PBMCs suggests peripheral suppressive populations (e.g., myeloid-derived suppressor cells, MDSCs) may modulate efficacy. Strengths include multimodal functional and spatial readouts and clear translational relevance. Limitations are small cohort size and absence of systemic physiology. Prospective validation linking *ex vivo* readouts to clinical responses, TCR clonotype tracking, single-cell profiling, and interrogation of myeloid suppression are warranted to establish clinical utility for personalizing immunotherapy in colorectal cancer (CRC).

## 3D organoid models bring new hope for osteoarthritis diagnosis and treatment (edited by Talita Glaser)

Jiao et al. provide a concise review of osteochondral organoids as transformative platforms for osteoarthritis (OA) research, highlighting their capacity to recapitulate cartilage–bone interfaces, multicellular crosstalk, and pathomimetic processes relevant to disease progression and repair. The authors synthesize advances in cell sourcing (MSCs, iPSC-derived progenitors), engineered matrices (natural, synthetic, gradient and DNA-based hydrogels), and bio fabrication (3D/4D bioprinting, microfluidics, integrated printing-chip systems) that enable graded osteochondral architectures and functional maturation. They emphasize applications in mechanistic modeling, gene-edited disease phenotypes, high-throughput drug screening, and regenerative implantation while underscoring persistent translational barriers: vascularization, immune compatibility, reproducibility under GMP, and safety concerns (e.g., residual pluripotent cells). Jiao et al. advocate interdisciplinary integration—organ-on-chip platforms, multi-omics, and AI-driven design and quality control—to generate standardized, patient-specific models that bridge preclinical discovery and clinical translation. Overall, the review positions osteochondral organoids as a promising route toward precision OA therapeutics, contingent on resolving key bioengineering and regulatory challenge.

## Organoids in cancer therapeutics—Preserving tumor heterogeneity for personalized treatment (edited by Uwe Ritter)

Xin-Yi et al. (a) and Xin-Yi et al. (b) review organoid technology as a promising transformative platform for cancer research and precision therapy. As three-dimensional, patient-derived mini-tissues, tumor organoids preserve genetic diversity and molecular signatures of primary tumors, enabling faithful modeling of intratumoral heterogeneity, tumor progression and metastasis. Methodologically, submerged, microfluidic and air–liquid-interface systems facilitate study of cell–cell interactions, immune responses and drug effects; living biobanks of patient-derived organoids (PDOs) provide platforms for personalized drug screening, target identification and combinatorial therapy testing. Clinically promising applications include prediction of drug sensitivity and evaluation of immunotherapies (e.g., CAR-T co-cultures). Key limitations: remain incomplete vascularization and stromal reconstruction, limited reproducibility, transient immune components, and ethical and scalability challenges. Integration with multi-omics, organ-on-chip and bioprinting technologies and optimized co-culture protocols may overcome these barriers and accelerate clinical translation. Overall, organoids offer a powerful but still maturing foundation for precision oncology and patient-centered therapeutic strategies.

## Future perspectives

The implementation of organoid co-culture models in clinical approaches will require the coordination across advances in methods, validation, engineering, and regulation. Central priorities include the adoption of harmonized reporting standards for co-culture protocols and functional endpoints to strengthen reproducibility and enable robust cross-study comparisons; prospective, adequately powered clinical studies to determine the predictive value of *ex vivo* organoid readouts for patient outcomes; and further bioengineering innovation, particularly in the development of modular vascular and immune compartments, to enhance culture longevity and more faithful model systemic-to-local immune dynamics. In parallel, the establishment of streamlined, automated, GMP-compatible platforms will be essential to reduce turnaround times, lower costs, and ensure consistency. Progress will be most effectively driven by interdisciplinary consortia that unite clinicians, bioengineers, bioinformaticians, immunologists, multi-omics experts, and regulatory stakeholders.

## Conclusion

This Research Topic illustrates that organoid co-culture systems are evolving from mechanistic experimental models toward translational platforms with real potential to inform precision medicine. The ability of these systems to recapitulate tissue architecture, spatial cellular heterogeneity, and multimodal function, position them as a promising frontier in biomedical research. Biological, engineering, and regulatory challenges still persist in constraining clinical translation. Fulfilling clinical impact will depend on standardized reporting, scalable bioengineering for vascular and immune integration, automated GMP-compatible manufacturing, prospective clinical validation, and harmonized analytic workflows, all pursued within collaborative networks that connect scientific and clinical expertise.

