# Correction: Editorial: Innovative organoid co-culture systems for enhanced precision medicine in cancer and beyond

**DOI:** 10.3389/fmed.2026.1927088

**Published:** 2026-08-07

**Authors:** Talita Glaser, Rupert Ecker, Uwe Ritter

**Affiliations:** 1 Department of Microbiology, Institute of Biomedical Sciences, University of São Paulo, São Paulo, Brazil; 2 Faculty of Health, School of Biomedical Sciences, Queensland University of Technology, Brisbane, QLD, Australia; 3 TissueGnostics GmbH, Vienna, Austria; 4 Chair for Immunology, University of Regensburg, Regensburg, Germany; 5 Leibniz Institute for Immunotherapy (LIT), Regensburg, Germany

**Keywords:** co-culture, *ex vivo* therapeutic response, organoid, personalized medicine, tumor microenvironment

Affiliation “Leibniz Institute for Immunotherapy (LIT), Regensburg, Germany” was omitted for author [Uwe Ritter]. This affiliation has now been added for author [Uwe Ritter].

Affiliation “Chair for Immunology, University of Regensburg, Regensburg, Germany” was erroneously given as “Department of Immunology, University of Regensburg, Regensburg, German”

The original version of this article has been updated.

